# Size and surface modification of amorphous silica particles determine their effects on the activity of human CYP3A4 *in vitro*

**DOI:** 10.1186/1556-276X-9-651

**Published:** 2014-12-02

**Authors:** Shunji Imai, Yasuo Yoshioka, Yuki Morishita, Tokuyuki Yoshida, Miyuki Uji, Kazuya Nagano, Yohei Mukai, Haruhiko Kamada, Shin-ichi Tsunoda, Kazuma Higashisaka, Yasuo Tsutsumi

**Affiliations:** 1 Laboratory of Toxicology and Safety Science, Graduate School of Pharmaceutical Sciences, Osaka University, 1-6 Yamadaoka, Suita, Osaka 565-0871, Japan; 2 Laboratory of Biopharmaceutical Research, National Institute of Biomedical Innovation, 7-6-8 Saitoasagi, Ibaraki, Osaka 567-0085, Japan; 3 Laboratory of Innovative Antibody Engineering and Design, Center for Drug Innovation and Screening, National Institute of Biomedical Innovation, 7-6-8 Saitoasagi, Ibaraki, Osaka 567-0085, Japan; 4 The Center for Advanced Medical Engineering and Informatics, Osaka University, 1-6 Yamadaoka, Suita, Osaka 565-0871, Japan

**Keywords:** Nanomaterials, Silica nanoparticles, Size, Surface modification, CYP3A4, Human liver microsomes

## Abstract

Because of their useful chemical and physical properties, nanomaterials are widely used around the world - for example, as additives in food and medicines - and such uses are expected to become more prevalent in the future. Therefore, collecting information about the effects of nanomaterials on metabolic enzymes is important. Here, we examined the effects of amorphous silica particles with various sizes and surface modifications on cytochrome P450 3A4 (CYP3A4) activity by means of two different *in vitro* assays. Silica nanoparticles with diameters of 30 and 70 nm (nSP30 and nSP70, respectively) tended to inhibit CYP3A4 activity in human liver microsomes (HLMs), but the inhibitory activity of both types of nanoparticles was decreased by carboxyl modification. In contrast, amine-modified nSP70 activated CYP3A4 activity. In HepG2 cells, nSP30 inhibited CYP3A4 activity more strongly than the larger silica particles did. Taken together, these results suggest that the size and surface characteristics of the silica particles determined their effects on CYP3A4 activity and that it may be possible to develop silica particles that do not have undesirable effects on metabolic enzymes by altering their size and surface characteristics.

## Background

The small size and high surface area of nanomaterials (which are defined as materials with at least one external dimension in the size range of 1 to 100 nm) give them useful properties such as unique chemical reactivity, heat conductivity, and ability to permeate tissues. Therefore, nanomaterials are expected to be used for applications in many fields [1,2]. In particular, amorphous silica nanoparticles are among the most widely used nanomaterials because of their comparatively low cost, their straightforward synthesis, and the ease with which their surfaces can be modified [3]. Silica nanoparticles are already widely used in cosmetics, food, and medicines [4-6]. Therefore, collecting information about the safety of silica nanoparticles is important [7,8]. In previous work, we found that they can penetrate the skin and enter various tissues [9] and that at high doses, they are more likely to induce consumptive coagulopathy and liver damage than do silica microparticles [10].

Because silica nanoparticles are used in food and drugs, their effects on metabolic enzymes such as cytochrome P450s (CYPs) are of particular interest. Xenobiotics such as drugs are metabolized by CYPs which are expressed at the highest levels in the liver. Cytochrome P450 3A4 (CYP3A4) is the most abundant CYP isozyme expressed in human liver tissue and is involved in the metabolism of approximately half of the drugs in use [11,12]. Drugs, some foods and beverages, and various chemicals such as those in cigarette affect the activity of CYPs. For example, ketoconazole, cyclosporine A, ritonavir, and grapefruit juice inhibit CYP3A4 activity and thus can lead to side effects when taken with drugs metabolized by CYP3A4 [13,14]. In contrast, rifampicin and St. John’s wort induce CYP3A4 and thus reduce the efficacy of some drugs that undergo CYP3A4-dependent metabolism [13,15]. Nanomaterials have also been reported to affect CYP3A4 activity. For example, nonmetallic carboxyl polystyrene nanoparticles (20 nm) inhibit the activity of CYP3A4 in microsomes isolated from baculovirus-infected cells expressing wild-type CYP3A4 [16], and silver nanoparticles dose-dependently decrease the amount of 6β-hydroxytestosterone, which is generated mainly by CYP3A4, in human liver microsomes (HLMs) [17].

Considering that silica nanoparticles are already used in foods and medicines, their effects on CYPs must be thoroughly explored. Silica nanoparticles are reported to be distributed to the liver after dermal, oral, intranasal, and intravenous administration [9,18,19]. In addition, we previously demonstrated that 70-nm silica nanoparticles are localized in the cytoplasm, which contains many enzymes related to metabolism such as CYPs [9]; therefore, silica nanoparticles have the opportunity to react with CYPs. Furthermore, Nishimori et al. and Li et al. showed that when administered to mice together, 70-nm silica nanoparticles and some drugs increased the toxicity on the liver relative to that observed when either is administered alone in mice [20,21]. These results suggest that silica nanoparticles may affect the activity of CYPs, but these potential effects have not been evaluated. In addition, little information is available about the effects of the size and surface characteristics of nanomaterials on CYP3A4 activity. In this study, we examined CYP3A4 activity in human hepatocellular carcinoma cells (HepG2) and in HLMs exposed to silica particles with various sizes and surface modifications.

## Methods

### Silica particles

Silica nanoparticles with diameters of 30 and 70 nm (nSP30 and nSP70, respectively), conventional silica microparticles with diameters of 300 and 1,000 nm (mSP300 and mSP1000, respectively), and nSP30 and nSP70 modified with carboxyl groups (nSP30-C and nSP70-C, respectively) or amine groups (nSP30-N and nSP70-N, respectively) were purchased from Micromod Partikeltechnologie GmbH (Friedrich-Barnewitz, Rostock, Germany). The silica particles were suspended in water, and the suspensions were stored at room temperature. Immediately prior to use, they were sonicated for 5 min and then vortexed for 1 min.

### Physicochemical examination of the silica preparations

Silica particles were diluted to 0.1 or 0.2 mg/mL with ultrapure water or Dulbecco’s modified Eagle’s medium (Wako Pure Chemical Industries, Osaka, Japan) supplemented with 10% fetal calf serum (FCS) and 1% antibiotic-antimycotic mix stock solution (Ab) (Gibco, Carlsbad, CA, USA), and the average particle size and surface charge (zeta potential) were determined using a Zetasizer Nano-ZS (Malvern Instruments Ltd., Malvern, UK). The size of silica particles were measured by dynamic light scattering. The surface charge was measured by laser Doppler electrophoresis.

### Reagents

Pooled HLMs (Xtreme 200) were obtained from XenoTech (Lenexa, KS, USA). Ketoconazole, a representative CYP3A4 inhibitor, was obtained from Wako Pure Chemical Industries (Osaka, Japan). Luciferin-isopropyl acetal (LIPA; Promega, Madison, WI, USA), which is metabolized specifically by CYP3A4 and releases luciferin, was used as a probe substrate to quantify CYP3A4 activity by luminescence after the reaction of LIPA with an ATP-luciferase reaction mixture [22].

### Evaluation of CYP3A4 activity in HLMs

The inhibitory effects of silica particles at various concentrations (2, 10, 50, 200, and 800 μg/mL) and ketoconazole (200 nmol/L) were determined with HLMs (20 μg/mL) in the presence of NADPH Regenerating System (Promega). The incubation mixtures, which consisted of silica particles, ketoconazole, 10 μmol/L LIPA, and HLMs in potassium phosphate buffer (15 μL, respectively), were pre-incubated for 10 min at 37°C, and then the enzymatic reactions were initiated by the addition of 15 μL of NADPH. In addition, to determine whether LIPA was physically bound to the silica particles, we also started the reaction by adding 15 μL of LIPA after preparing a mixture of the silica particles, HLMs, buffer, and NADPH. Next, to determine whether the silica particles were physically bound to microsome proteins, we centrifuged a mixture of silica particles, LIPA, HLMs, and buffer at 1,000 × *g* or 5,000 × *g* for 20 min and then added NADPH (15 μL) to the supernatant (45 μL). For each of these procedures, the reactions were terminated after 10 min of incubation by the addition of reconstitution buffer (60 μL). Each plate was incubated at room temperature for 20 min, and then the luminescence was read with a luminometer for 1 s per well.

### Cell culture

HepG2 cells were maintained in Dulbecco’s modified Eagle’s medium including 10% FCS and 1% Ab.

### Lactate dehydrogenase release assay in HepG2 cells

The lactate dehydrogenase (LDH) activity in HepG2 cells exposed to silica particles was determined with a commercial LDH cytotoxicity test (Wako Pure Chemical Industries) conducted according to the manufacturer’s instructions. In brief, HepG2 cells (1 × 10^5^ cells/well) were pre-cultured in 24-well plates for 24 h, the pre-culture medium was removed, and the cells were incubated with silica particles (25 to 200 μg/mL) for 48 h. Then, 50 μL of the supernatant was used for LDH analysis. Absorbance at 570 nm was measured with a spectrophotometer. The percentage of cellular survival was calculated by means of the following equation:

$$\mathrm{Cellular}\;\mathrm{survival}\;\left(\%\right)\;=\;100\;-\left[100\left(A–B\right)/\left(C\;–\;B\right)\right]$$

where *A* is the absorbance measured for a well treated with silica particles, *B* is the absorbance measured for an untreated well, and *C* is the absorbance measured for a well treated with 0.1% Triton X.

### Evaluation of CYP3A4 activity in HepG2 cells

HepG2 cells were treated with silica particles by means of the protocol described for the LDH release assay. After a 48-h incubation period, the incubation medium was aspirated, the cells were washed twice with phosphate-buffered saline, and 200 μL of LIPA (3 μmol/L) was added to each well. After 1 h, a 100-μL aliquot of culture medium including LIPA was transferred from each well to a 96-well opaque white luminometer plate at room temperature, and then 100 μL of Luciferin Detection Reagent was added to initiate the luminescence reaction. The plate was incubated at room temperature for 20 min, and then the luminescence was read for 1 s with a luminometer.

### Statistical analysis

All data are presented as mean ± SD. Differences were compared by means of Dunnett’s test. Differences between experimental groups and the control group were considered significant at *P* < 0.05.

## Results and discussion

To evaluate the effect of silica particles on CYP3A4 activity, we measured CYP3A4 activity of HLMs and HepG2 cells after treating with silica particles. To elucidate the influence of size and surface modification of silica nanoparticles on their effect for CYP3A4 activity, we used silica particles with various sizes and surface modifications.

### Physicochemical properties of silica particles

First, the particle size and surface charge of silica particles in water and medium were measured. The particle size and surface charge of several particles used in this study were reported in previous studies [9,23-26]. However, we measured these parameters again. Mean particle sizes of nSP30, nSP30-C, nSP30-N, nSP70, nSP70-C, nSP70-N, mSP300, and mSP1000 measured by dynamic light scattering method were 36.8 ± 0.3, 49.0 ± 1.7, 40.4 ± 0.9, 86.2 ± 2.7, 78.7 ± 0.3, 103 ± 0, 293.0 ± 2.7, and 1,253.3 ± 32.1 nm (in water), respectively, and 84.9 ± 1.9, 294.0 ± 45.0, 410.3 ± 48.2, 128.3 ± 2.3, 267.0 ± 28.6, 267.3 ± 2.1, 249.3 ± 24.0, and 1,083.3 ± 35.1 nm (in medium), respectively. The surface charge of nSP30, nSP30-C, nSP30-N, nSP70, nSP70-C, nSP70-N, mSP300, and mSP1000 measured by laser Doppler electrophoresis was -32.5 ± 1.4, -46.9 ± 1.8, -18.3 ± 1.9, -58.4 ± 0.2, -64.3 ± 1.9, -35.6 ± 1.1, -56.4 ± 0.7, and -72.4 ± 0.6 mV (in water), respectively, and -9.0 ± 1.0, -11.2 ± 0.2, -11.1 ± 1.1, -10.0 ± 0.1, -10.2 ± 0.5, -10.1 ± 1.0, -9.3 ± 0.5, and -10.0 ± 1.6 mV (in medium), respectively.

### Effects of silica particles on CYP3A4 activity in HLMs

**Figure 1:**
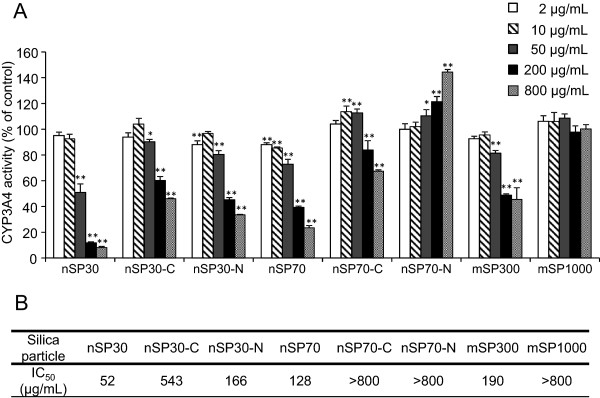
**Effect of silica particles on CYP3A4 activity in HLMs. (A)** Silica particles were incubated with HLMs for 10 min. Then, CYP3A4 activity was measured, and the percentage of CYP3A4 activity was calculated relative to the activity of sterile water as a control. Data are presented as mean ± SD for three independent determinations. **P < 0.01 and *P < 0.05 versus the control group (Dunnett’s test). **(B)** IC_50_ values for inhibition of CYP3A4 activity in HLMs by silica particles.

**Figure 2:**
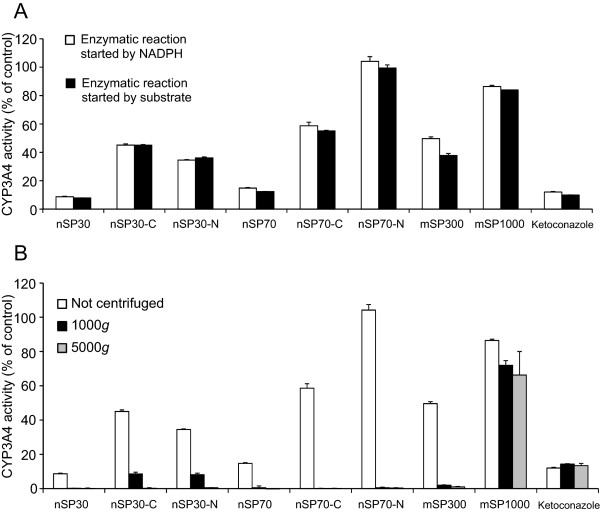
**Evaluation of the interaction between silica particles and a probe substrate or CYP3A4. (A)** Silica particles (200 μg/mL) or ketoconazole (200 nmol/L) was incubated with HLMs, and CYP3A4 activity was then measured in two ways: (1) the enzymatic reaction was started by the addition of NADPH after pre-incubation of the silica particles with the probe substrate, or (2) the enzymatic reaction was started by the addition of the probe substrate after pre-incubation of the silica particles with NADPH. **(B)** Mixtures of microsomes, the probe substrate, and silica particles were centrifuged at 1,000 × *g* or 5,000 × *g* for 20 min, or the mixtures were not centrifuged. The CYP3A4 activity of each supernatant was measured, and the percentage of CYP3A4 activity was calculated relative to the activity of sterile water as a control. Data are presented as mean ± SD for three independent determinations.

Silica particles at concentrations of 2, 10, 50, 200, and 800 μg/mL were incubated with HLMs, and inhibition constants (IC_50_) were determined (Figure 1). The activity of CYP3A4 decreased dose-dependently upon co-incubation with nSP30, nSP30-C, nSP30-N, nSP70, nSP70-C, or mSP300. In contrast, we observed no significant difference in the CYP3A4 activity of the mSP1000-treated group compared to that of the control group. For the unmodified silica particles, IC_50_ increased with increasing particle size. This result suggests that smaller silica particles have a greater potential to suppress CYP3A4 activity. In contrast, the IC_50_ values for nSP30 and nSP70 were lower than the values for nSP30-C, nSP30-N, and nSP70-C, indicating that surface modification changed the inhibitory potential of the particles. Surprisingly, we found that modification of nSP70 with amine groups resulted in increased CYP3A4 activity. Note, however, that differences in the size of nanomaterials reportedly affect their protein-binding mode [27]. Therefore, nSP30-N, which also has amino groups but did not activate CYP3A4, may interact with CYP3A4 differently than nSP70-N does.Next, we investigated the mechanism of the effects of silica nanoparticles on CYP3A4 activity. First, we considered the possibility that the silica particles bound to the substrate and prevented interaction of the substrate and the enzyme (Figure 2A). We compared CYP3A4 activity under the following two conditions: (1) the enzymatic reaction was started by the addition of NADPH, which causes microsomal oxidation, after pre-incubation of the silica particles with a probe substrate, and (2) the enzymatic reaction was started by the addition of the probe substrate after pre-incubation of the silica particles with NADPH. Instead of silica particles, we also used ketoconazole which suppresses CYP3A4 activity without affecting the substrate. For all the silica particles and ketoconazole, the CYP3A4 activity was almost exactly the same under the two conditions, indicating that the silica particles did not affect the substrate-enzyme interaction.

To determine whether the silica particles physically bound to microsome proteins, we centrifuged mixtures of microsomes, substrate, and silica particles and then measured the CYP3A4 activity in the supernatant (Figure 2B). We found that the CYP3A4 activity in groups treated with silica particles was dramatically lower in the centrifugation group compared to the uncentrifuged control, except in the case of the group treated with mSP1000 and ketoconazole. Microsome proteins and ketoconazole are not usually precipitated by centrifugation at either 1,000 × *g* or 5,000 × *g*. Therefore, these results suggest that microsome proteins bound to the silica nanoparticles and to mSP300 to form complexes that were heavy enough to be precipitated by centrifugation at 1,000 × *g* or 5,000 × *g*. Thus, we suggest that silica nanoparticles and mSP300 physically bound to microsome proteins and affected the CYP3A4 activity in HLMs. Bertoli et al. suggested the possibility that silica-coated magnetic 50-nm nanoparticles bind to CYPs in cells [28]. Therefore, mSP300 and the silica nanoparticles (except nSP70-N) may have bound to CYP3A4 and blocked its substrate-binding site or may have converted CYP3A4 to an inactive conformation, whereas nSP70-N bound to CYP3A4 and changed it to an active conformation.

### Cytotoxicity of silica particles to HepG2 cells and CYP3A4 activity in HepG2 cells

**Figure 3:**
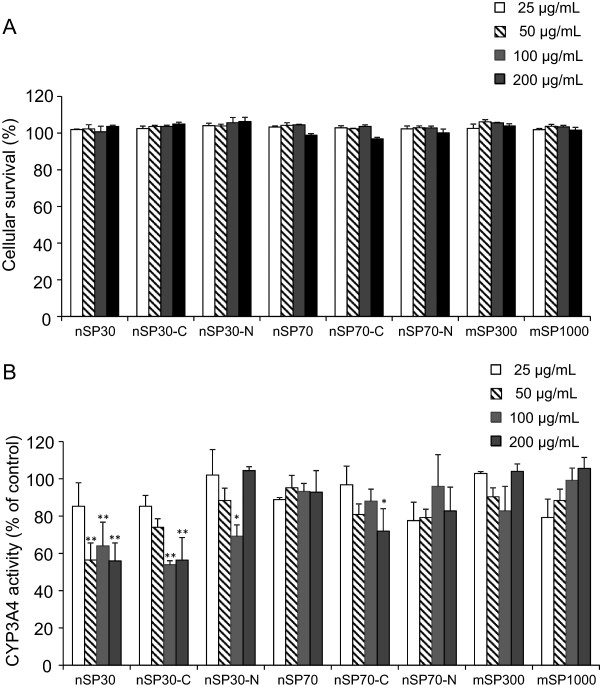
**Effect of silica particles on membrane damage and CYP3A4 activity in HepG2 cells.** HepG2 cells were incubated with silica particles for 48 h. **(A)** An LDH release assay was conducted with the supernatant, and the percentage of surviving cells was calculated. **(B)** CYP3A4 activity in HepG2 cells was measured, and CYP3A4 activity was calculated relative to that of the culture medium as a control. Data are presented as mean ± SD for three independent cultures. **P < 0.01 and *P < 0.05 versus control group (Dunnett’s test).

To examine the influence of silica particles on CYP3A4 activity in hepatocytes, we evaluated cellular survival (Figure 3A) and CYP3A4 activity (Figure 3B) in HepG2 cells after incubation with silica particles for 48 h. The survival rates in the groups treated with silica particles at concentrations of 25, 50, 100, and 200 μg/mL were almost 100%, indicating that none of the particles induced membrane damage in HepG2 cells under our experimental conditions (Figure 3A). In contrast, treatment with nSP30 at 50, 100, or 200 μg/mL, treatment with nSP30-C at 100 or 200 μg/mL, treatment with nSP30-N at 100 μg/mL, or treatment with nSP70-C at 200 μg/mL resulted in significantly lower CYP3A4 activity compared with the activity in the control group, whereas treatment with nSP70, nSP70-N, mSP300, or mSP1000 had no effect on CYP3A4 activity. These results suggest that smaller silica particles had greater potential to suppress CYP3A4 activity in cells. We confirmed that nSP30-N were aggregated at 200 μg/mL (mean particle size in medium, 410.3 ± 48.2 nm) compared to those at 100 μg/mL (mean particle size in medium, 121.0 ± 15.4 nm). That may be the reason why the treatment of nSP30-N at 200 ug/mL did not decrease CYP3A4 activity.

Both the HepG2 assay and the HLM assay showed similar results with regard to the relationship between particle size and CYP3A4 inhibition activity. However, the two assays showed slightly different results with regard to the relationship between surface modification and CYP3A4 activity. In HepG2 cells (Figure 3B), the nSP70-C-treated group showed significantly inhibited CYP3A4 activity compared to the control group at a concentration at which nSP70 did not inhibit the activity (200 μg/mL), and nSP30-C showed almost the same CYP3A4 inhibition activity as nSP30. In contrast, the HLM assay showed that nSP70 and nSP30 inhibited CYP3A4 more strongly than did nSP70-C and nSP30-C, respectively (Figure 1). The explanation for the difference between the unmodified silica nanoparticles and carboxyl-modified nanoparticles remains to be determined. However, Chung et al. showed that the surface charge of mesoporous silica particles could mediate cellular uptake rate or even cellular uptake route of the particles [29]. Therefore, change of surface charge by carboxyl modification may affect silica nanoparticles’ cellular uptake. In fact, Ekkapongpisit et al. showed that carboxyl modification of 50-nm mesoporous silica particles changes their cellular uptake [30], and we have also found that nSP70 and nSP70-C have different intracellular localizations [25]. Thus, differences in cellular uptake or intracellular localization between unmodified and carboxyl-modified silica nanoparticles may explain the different results observed in cells and under conditions where the particles have direct access to microsomes.

## Conclusions

We report for the first time that silica particles can either inhibit or activate CYP3A4 activity *in vitro*. We showed that smaller particles have a greater potential to inhibit CYP3A4 activity than larger particles and that surface modification of silica particles could change their effects on CYP3A4 activity. Our results suggest that optimization of the size and surface modification of silica particles will contribute to the development of safer applications of silica nanoparticles.

## Abbreviations

CYP3A4: cytochrome P450 3A4; HLMs: human liver microsomes; LDH: lactate dehydrogenase; LIPA: luciferin-isopropyl acetal.

## Competing interests

The authors declare that they have no competing interests.

## Authors’ contributions

SI, YY, and TY designed the study. SI, TY, and MU performed the experiments. SI, YY, YuM, and TY collected and analyzed the data. SI, YY, and YuM wrote the manuscript. KN, YoM, HK, ST, and KH, provided technical support and conceptual advice. YT supervised the project. All authors discussed the results and commented on the manuscript. All authors read and approved the final manuscript.

## References

[B1] Cormode DP Jarzyna PA Mulder WJ Fayad ZA Modified natural nanoparticles as contrast agents for medical imaging Adv Drug Deliv Rev 2010 62 329 338 19900496 10.1016/j.addr.2009.11.005 PMC2827667

[B2] Kaur IP Agrawal R Nanotechnology: a new paradigm in cosmeceuticals Recent Pat Drug Deliv Formul 2007 1 171 182 19075884 10.2174/187221107780831888

[B3] Fadeel B Garcia-Bennett AE Better safe than sorry: understanding the toxicological properties of inorganic nanoparticles manufactured for biomedical applications Adv Drug Deliv Rev 2010 62 362 374 19900497 10.1016/j.addr.2009.11.008

[B4] Wang H Du LJ Song ZM Chen XX Progress in the characterization and safety evaluation of engineered inorganic nanomaterials in food Nanomedicine 2013 8 2007 2025 24279490 10.2217/nnm.13.176

[B5] Mody KT Popat A Mahony D Cavallaro AS Yu C Mitter N Mesoporous silica nanoparticles as antigen carriers and adjuvants for vaccine delivery Nanoscale 2013 5 5167 5179 23657437 10.1039/c3nr00357d

[B6] Ciriminna R Sciortino M Alonzo G Schrijver A Pagliaro M From molecules to systems: sol–gel microencapsulation in silica-based materials Chem Rev 2011 111 765 789 20726523 10.1021/cr100161x

[B7] Peters R Kramer E Oomen AG Rivera ZE Oegema G Tromp PC Fokkink R Rietveld A Marvin HJ Weigel S Peijnenburg AA Bouwmeester H Presence of nano-sized silica during in vitro digestion of foods containing silica as a food additive ACS Nano 2012 6 2441 2451 22364219 10.1021/nn204728k

[B8] Dekkers S Krystek P Peters RJ Lankveld DP Bokkers BG van Hoeven-Arentzen PH Bouwmeester H Oomen AG Presence and risks of nanosilica in food products Nanotoxicology 2011 5 393 405 20868236 10.3109/17435390.2010.519836

[B9] Nabeshi H Yoshikawa T Matsuyama K Nakazato Y Matsuo K Arimori A Isobe M Tochigi S Kondoh S Hirai T Akase T Yamashita T Yamashita K Yoshida T Nagano K Abe Y Yoshioka Y Kamada H Imazawa T Itoh N Nakagawa S Mayumi T Tsunoda S Tsutsumi Y Systemic distribution, nuclear entry and cytotoxicity of amorphous nanosilica following topical application Biomaterials 2011 32 2713 2724 21262533 10.1016/j.biomaterials.2010.12.042

[B10] Nabeshi H Yoshikawa T Matsuyama K Nakazato Y Arimori A Isobe M Tochigi S Kondoh S Hirai T Akase T Yamashita T Yamashita K Yoshida T Nagano K Abe Y Yoshioka Y Kamada H Imazawa T Itoh N Kondoh M Yagi K Mayumi T Tsunoda S Tsutsumi Y Amorphous nanosilicas induce consumptive coagulopathy after systemic exposure Nanotechnology 2012 23 045101 22214761 10.1088/0957-4484/23/4/045101

[B11] Guengerich FP Cytochrome P-450 3A4: regulation and role in drug metabolism Annu Rev Pharmacol Toxicol 1999 39 1 17 10331074 10.1146/annurev.pharmtox.39.1.1

[B12] Liu YT Hao HP Liu CX Wang GJ Xie HG Drugs as CYP3A probes, inducers, and inhibitors Drug Metab Rev 2007 39 699 721 18058330 10.1080/03602530701690374

[B13] Scheen AJ Drug-drug and food-drug pharmacokinetic interactions with new insulinotropic agents repaglinide and nateglinide Clin Pharmacokinet 2007 46 93 108 17253883 10.2165/00003088-200746020-00001

[B14] Srinivas NR Is there a place for drug combination strategies using clinical pharmacology attributes?–review of current trends in research Curr Clin Pharmacol 2009 4 220 228 19500074 10.2174/157488409789375285

[B15] Rahimi R Abdollahi M An update on the ability of St. John’s wort to affect the metabolism of other drugs Expert Opin Drug Metab Toxicol 2012 8 691 708 22606944 10.1517/17425255.2012.680886

[B16] Frohlich E Kueznik T Samberger C Roblegg E Wrighton C Pieber TR Size-dependent effects of nanoparticles on the activity of cytochrome P450 isoenzymes Toxicol Appl Pharmacol 2010 242 326 332 19909766 10.1016/j.taap.2009.11.002

[B17] Lamb JG Hathaway LB Munger MA Raucy JL Franklin MR Nanosilver particle effects on drug metabolism in vitro Drug Metab Dispos 2010 38 2246 2251 20861156 10.1124/dmd.110.035238

[B18] Fu C Liu T Li L Liu H Chen D Tang F The absorption, distribution, excretion and toxicity of mesoporous silica nanoparticles in mice following different exposure routes Biomaterials 2013 34 2565 2575 23332175 10.1016/j.biomaterials.2012.12.043

[B19] Yoshida T Yoshioka Y Tochigi S Hirai T Uji M Ichihashi K Nagano K Abe Y Kamada H Tsunoda S Nabeshi H Higashisaka K Yoshikawa T Tsutsumi Y Intranasal exposure to amorphous nanosilica particles could activate intrinsic coagulation cascade and platelets in mice Part Fibre Toxicol 2013 10 41 23958113 10.1186/1743-8977-10-41 PMC3751833

[B20] Li X Kondoh M Watari A Hasezaki T Isoda K Tsutsumi Y Yagi K Effect of 70-nm silica particles on the toxicity of acetaminophen, tetracycline, trazodone, and 5-aminosalicylic acid in mice Pharmazie 2011 66 282 286 21612156

[B21] Nishimori H Kondoh M Isoda K Tsunoda S Tsutsumi Y Yagi K Influence of 70 nm silica particles in mice with cisplatin or paraquat-induced toxicity Pharmazie 2009 64 395 397 19618677

[B22] Li AP Evaluation of luciferin-isopropyl acetal as a CYP3A4 substrate for human hepatocytes: effects of organic solvents, cytochrome P450 (P450) inhibitors, and P450 inducers Drug Metab Dispos 2009 37 1598 1603 19451401 10.1124/dmd.109.027268

[B23] Yamashita K Yoshioka Y Higashisaka K Mimura K Morishita Y Nozaki M Yoshida T Ogura T Nabeshi H Nagano K Abe Y Kamada H Monobe Y Imazawa T Aoshima H Shishido K Kawai Y Mayumi T Tsunoda S Itoh N Yoshikawa T Yanagihara I Saito S Tsutsumi Y Silica and titanium dioxide nanoparticles cause pregnancy complications in mice Nat Nanotechnol 2011 6 321 328 21460826 10.1038/nnano.2011.41

[B24] Nabeshi H Yoshikawa T Matsuyama K Nakazato Y Tochigi S Kondoh S Hirai T Akase T Nagano K Abe Y Yoshioka Y Kamada H Itoh N Tsunoda S Tsutsumi Y Amorphous nanosilica induce endocytosis-dependent ROS generation and DNA damage in human keratinocytes Part Fibre Toxicol 2011 8 1 21235812 10.1186/1743-8977-8-1 PMC3030505

[B25] Nabeshi H Yoshikawa T Arimori A Yoshida T Tochigi S Hirai T Akase T Nagano K Abe Y Kamada H Tsunoda S Itoh N Yoshioka Y Tsutsumi Y Effect of surface properties of silica nanoparticles on their cytotoxicity and cellular distribution in murine macrophages Nanoscale Res Lett 2011 6 93 21711578 10.1186/1556-276X-6-93 PMC3212243

[B26] Higashisaka K Yoshioka Y Yamashita K Morishita Y Fujimura M Nabeshi H Nagano K Abe Y Kamada H Tsunoda S Yoshikawa T Itoh N Tsutsumi Y Acute phase proteins as biomarkers for predicting the exposure and toxicity of nanomaterials Biomaterials 2011 32 3 9 20864168 10.1016/j.biomaterials.2010.08.110

[B27] Deng ZJ Liang M Toth I Monteiro MJ Minchin RF Molecular interaction of poly(acrylic acid) gold nanoparticles with human fibrinogen ACS Nano 2012 6 8962 8969 22998416 10.1021/nn3029953

[B28] Bertoli F Davies GL Monopoli MP Moloney M Gun’ko YK Salvati A Dawson KA Magnetic nanoparticles to recover cellular organelles and study the time resolved nanoparticle-cell interactome throughout uptake Small 2014 10 3307 3315 24737750 10.1002/smll.201303841

[B29] Chung TH Wu SH Yao M Lu CW Lin YS Hung Y Mou CY Chen YC Huang DM The effect of surface charge on the uptake and biological function of mesoporous silica nanoparticles in 3 T3-L1 cells and human mesenchymal stem cells Biomaterials 2007 28 2959 2966 17397919 10.1016/j.biomaterials.2007.03.006

[B30] Ekkapongpisit M Giovia A Follo C Caputo G Isidoro C Biocompatibility, endocytosis, and intracellular trafficking of mesoporous silica and polystyrene nanoparticles in ovarian cancer cells: effects of size and surface charge groups Int J Nanomedicine 2012 7 4147 4158 22904626 10.2147/IJN.S33803 PMC3418080

